# Complex patterns and determinants of regional multiple chronic conditions across the United States

**DOI:** 10.1093/pnasnexus/pgae513

**Published:** 2024-11-14

**Authors:** Yanqing Xu, Ming Yan, Cong Fu, Wei Xu, Yan Liu, Yuchen Li

**Affiliations:** School of Remote Sensing and Information Engineering, Wuhan University, Wuhan, Hubei 430079, China; School of Remote Sensing and Information Engineering, Wuhan University, Wuhan, Hubei 430079, China; School of Remote Sensing and Information Engineering, Wuhan University, Wuhan, Hubei 430079, China; Health Management Center, Renmin Hospital of Wuhan University, Wuhan, Hubei 430060, China; School of Remote Sensing and Information Engineering, Wuhan University, Wuhan, Hubei 430079, China; School of Geography, University of Leeds, Leeds LS2 9JT, United Kingdom; MRC Epidemiology Unit, School of Clinical Medicine, University of Cambridge, Cambridge CB2 0QQ, United Kingdom

**Keywords:** noncommunicable chronic diseases, multiple chronic conditions, risk factors, public health, network analysis

## Abstract

Noncommunicable chronic diseases (NCDs) are a rapidly growing global public health concern, posing substantial challenges to healthcare systems. The presence of multiple (≥2) chronic conditions (MCC) exacerbates these challenges. In this study, we constructed an integrated MCC network to comprehensively evaluate the impact of NCD prevalence and associated factors on MCC patterns. We identified four distinct MCC patterns, each with its unique set of associated risk factors. Firstly, we found that race, sedentary lifestyles, and smoking habits were significant contributors to the co-occurrence of diabetes, chronic kidney disease, and cancer. Secondly, smoking habits and mental health were identified as risk factors associated with the clusters of high cholesterol, hypertension, coronary heart disease, and arthritis. Furthermore, the comorbidity of chronic obstructive pulmonary disease and asthma was affected by socioeconomic status, smoking habits, and educational attainment, and a noteworthy reciprocal relationship existed between these two MCC combinations. Thirdly, the combination of asthma and obesity is associated with risk factors such as mental health, smoking habits, sedentary lifestyles, and binge drinking behaviors. Finally, the pattern of depression-stroke comorbidity was influenced by risk factors including mental health, age, and sleep duration. Our findings hold valuable implications for healthcare system optimization, offering a pathway to mitigate the escalating burden of NCDs. Additionally, they provide a foundation for scientific strategies aimed at the joint prevention and management of these complex conditions, ultimately enhancing public health and safety on a global scale.

**Significance Statement**

In addressing the burgeoning global burden of noncommunicable chronic diseases, this study unveils four distinct patterns of regional multiple chronic conditions (MCC) in the United States, driven by various risk factors. Harnessing data from 12 diseases and 10 influencing elements, our findings expand knowledge on disease interplay, emphasizing factors like race, lifestyle choices, mental health, and socioeconomic status. This comprehensive outlook, which classifies disease roles within MCC frameworks, illuminates pathways for healthcare optimization. It paves the way for holistic strategies targeting prevention and management, advancing public health endeavors globally.

## Introduction

Over the past two decades, the primary causes of disease mortality and morbidity have shifted from infectious diseases to noncommunicable diseases (NCDs), including cardiovascular disease (CVD), kidney disease, and diabetes, among others. Their emergence poses a significant public health challenge, affecting both developed and developing countries while also imposing a considerable economic burden on healthcare systems and societies (1). Moreover, NCDs exhibit a tendency to co-occur, and their consequences on various health outcomes may vary based on a multitude of factors including an individual's sociodemographic characteristics, specific combinations of symptoms, and the presence of other health-related issues (2).

According to data from the 2018 National Health Interview Survey, approximately 27.2% of American adults experienced the burden of two or more NCDs (3). This phenomenon of multimorbidity is prevalent in global health, with its complexity arising from interactions among different NCDs and the influence of social, environmental, and economic factors (4, 5).

From the perspective of public health surveillance, obtaining accurate population-level multimorbidity data necessitates access to comprehensive medical records for individuals in the region. In order to safeguard patient privacy and mitigate data collection demands, we emphasize the concept of multiple chronic conditions (MCC) at the population level, which deviates from the conventional concept of MCC that typically refers to an individual experiencing the concurrent presence of two or more NCDs (6). We focus on the simultaneous prevalence of different sets of NCDs in a geographical unit. This regional research can use statistical data to monitor hotspots for prioritization in clinical practice, medical resource allocation, and health service distribution (7).

The American Geriatrics Society underscored the paramount importance of identifying conditions that commonly co-occur back in 2012 (8). Recognizing patterns of MCC and their associated risk factors is pivotal, as it not only helps curb the spread of NCDs but also optimizes healthcare services and addresses determinants of health. Compared with a single NCD, MCC carry a higher risk of mortality, increased medical costs, functional decline, disability, and a lower quality of life (9, 10). If a NCD heightens the risk of another, interventions such as lifestyle adjustments, early screenings, or specific management can be implemented. For example, there is a well-established reciprocal relationship between CVD and type 2 diabetes (11). People with diabetes are at a higher risk of developing CVD, and having CVD increases the risk of diabetes-related complications. Therefore, regular medical checkups for individuals with diabetes help in the early detection of any cardiovascular risk factors or complications. Moreover, understanding NCD interactions aids healthcare providers in formulating more efficient and precisely targeted treatment and prevention strategies (12), resulting in better health outcomes and improved quality of life for individuals with NCDs. Additionally, a comprehension of multidimensional factors contributing to MCC is crucial for tackling health disparities. Previous studies have reported that NCDs are affected by a variety of biological, psychological, and socioeconomic factors (4). Therefore, policymakers and healthcare organizations can work toward reducing health inequities and promoting health for all populations by investigating the synergistic effects of diverse risk factor variables concerning the prevalence of NCDs across these dimensions.

Some prior pieces of research have delved into the exploration of clustering patterns among MCC and combinations of NCDs due to their high-prevalence and common risk factors. In terms of investigating MCC arising from connections between different NCDs, one study, conducted in the United Kingdom among middle-aged and elderly individuals, employed clustering analysis, and association rule mining methods, revealing three clusters and 30 disease combinations. Conditions like diabetes, hypertension, and asthma were central to several disease groups (13). Additionally, an expert workshop sponsored by the National Institutes of Health summarized four models for assessing the existence and patterns of MCC. These models encompass classification and regression trees, qualifying comorbidity sets, the multimorbidity index, and the application of omics to network medicine (14). Among these models, network analysis is widely applied to identify clusters of highly connected nodes, enabling the discernment of NCDs with highly comorbid associations (15, 16). In terms of investigating MCC arising from shared risk factors, a study in India utilized a multinomial logistic regression model and discovered that adults with risk factors like alcohol consumption, overweight, and central obesity exhibited the highest prevalence of MCC (10). Moreover, a study employed logistic regression analysis to assess disparities in MCC occurrence among various populations, revealing that gender and economic status can influence the risk of MCC (17).

However, the studies mentioned above have provided limited consideration for the potential joint influence of both factors. The analysis of MCC patterns necessitates a comprehensive evaluation of the structural characteristics between NCDs and a thorough investigation into the interaction processes between MCC structures and risk factors. Traditional statistical methods have limitations in recognizing MCC patterns because of the inability to utilize characteristic information from multiple coexisting NCDs. In contrast, network analysis provides the opportunity to display the complex and variable correlations among MCC. Additionally, it can be used to evaluate the significance of NCDs and to apply community detection algorithms that can identify closely interconnected groups within the network. In summary, network analysis proves instrumental in unveiling concealed organizational structures and extracting MCC patterns.

In our current study, we undertook a nationwide ecological analysis utilizing county-level population health outcome data from 2020, focusing on the prevalence of different sets of NCDs in the United States. Our research aims to explore the interrelationships among these NCDs in terms of chronic disease prevalence across different geographical regions. A stronger correlation between two NCDs indicates a greater affinity in that region, suggesting a significantly increased risk for the population to develop such comorbid conditions (7). Several studies have used ecological designs to explore factors linked with MCC at the population level. Eun found that higher rates of crime, severe poverty, and elevated unemployment are associated with increased prevalence of arthritis, asthma, diabetes, heart disease, obesity, and stroke, using statistical data at the census tract level in Memphis, TN (7). Ace investigated the impact of four social determinants of health (namely, asthma, kidney disease, smoking, and food stamps) on length of life and quality of life in a population through a large dataset of census tracts in the United States (18). Emeka identified a positive correlation between the incidence of MCC and the aggregate social care expenditure by local authorities, based on a regional analysis (19). Young-Rock demonstrated a link between regional-level measures and CVD outcomes and suggested that seven social determinants (namely, minority, poverty, no high school diploma, grocery store ratio, fast-food restaurant ratio, after-tax soda price, and primary care physician supply) should be considered when assessing CVD mortality (20).

We undertook a nationwide ecological analysis employing network analysis to construct two distinct networks: one depicting disease–disease associations and the other illustrating disease-influencer associations. This approach allows us to visually and clearly depict the functions and interactions of contributors affecting NCDs. These networks were subsequently merged into an integrated MCC network, followed by a network analysis to identify MCC patterns. Furthermore, we have classified NCDs based on their roles in the MCC patterns. This integrated MCC network serves as a valuable tool for considering the impact of NCD prevalence and risk factors on MCC. The primary objectives of this study are as follows: (ⅰ) to explore the relationships among the prevalence of 12 NCDs in 3,143 US counties; (ⅱ) to analyze the correlations between risk factors and disease prevalence from four dimensions: biological, psychological, socioeconomic, and behavioral; (ⅲ) to identify regional MCC patterns and explore potential factors shaping these MCC combinations using county-level aggregated data; (ⅳ) to classify these NCDs based on their roles in MCC patterns. We anticipate that the findings from this study will foster further research into the role of NCDs in MCC patterns. These results will contribute to a deep understanding of the complexities of regional MCC, which is essential for exploring innovative prevention strategies and ultimately reducing the high incidence of NCDs.

## Results

### Characteristics of NCDs and their contributing factors

**Fig. 1.**
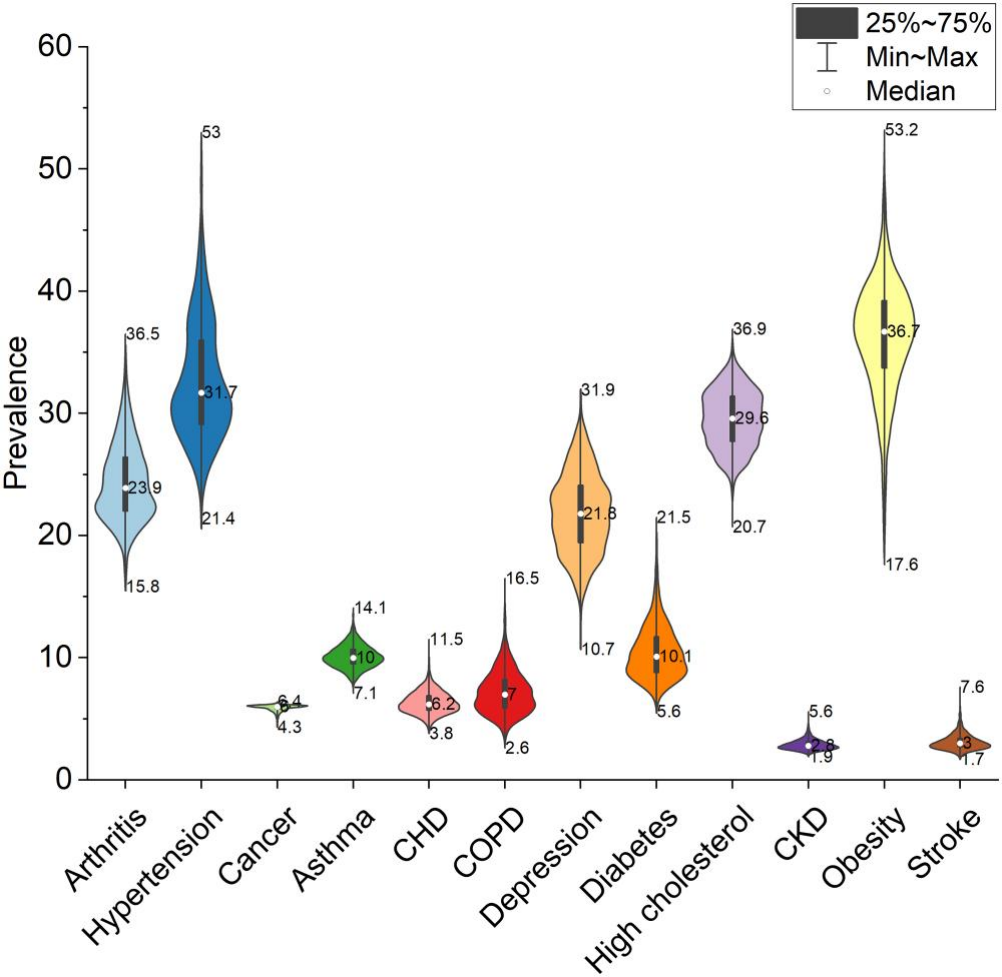
The age-adjusted distribution of the prevalence of NCDs among adults aged 18 years and older in 2020.

Figure 1 illustrates the distribution of age-adjusted prevalence data across counties for the 12 NCDs under analysis. We present the data in percentage values, including median, range statistics, and probability density information. The median prevalence rates for obesity (prevalence = 36.7) and hypertension (prevalence = 31.7) both exceed 30%. However, there is a significant imbalance in their values across different counties, with a substantial difference of 35.6% for obesity and 31.6% for hypertension. The prevalence of obesity is ∼36% in most areas, while the prevalence of hypertension is bimodal. The median prevalence rates for arthritis (prevalence = 23.9), depression (prevalence = 21.8), and high cholesterol (prevalence = 29.6) are in the range of 20 to 30%. Compared with high cholesterol, there is a wider variation in the prevalence distribution of arthritis and depression across counties, with values of 20.7 and 21.2%, respectively. Only the prevalence of arthritis shows a bimodal distribution, whereas the probability of high cholesterol values in the range of 25 to 30% is relatively consistent. Most NCDs have a prevalence rate of <20% across counties, including cancer, asthma, coronary heart disease (CHD), chronic obstructive pulmonary disease (COPD), diabetes, chronic kidney disease (CKD), and stroke. Among them, CKD (prevalence = 2.8) and stroke (prevalence = 3) have the lowest median prevalence rates. COPD and diabetes are the only NCDs that show a prevalence distribution variation of more than 10% across counties, with values of 13.9 and 15.9%, respectively. Meanwhile, the prevalence of other NCDs is concentrated within a relatively narrow range in most counties.

Table 1 displays the composition and descriptive statistical characteristics of the risk factors, including mean, variance, and maximum and minimum values. The numbers indicate the percentage of the affected population relative to the total population. Among the risk factors, “Minority,” “Impoverishment,” “Smoking,” and “Sedentary behavior” exhibit mean value ranging from 20 to 30%, specifically 24.25, 24.49, 20.04, and 25.71%, respectively. The highest mean among these factors is “Sleep deficiency” at 34.47%, while the lowest mean is “Lacking fundamental education” at 12.40%. The proportion of “Minority” exhibits a marked variation (20.22) among counties, spanning from a value (0) to the highest value (99). However, the variances of the other factors are all <10.

**Table 1.** Characteristics of explanatory variables in 2020.

Theme	Explanatory variable	Description	Mean	SD	Min	Max
Demographic factors	Minority	Percentage minority (all persons except non-Hispanic Whites) estimate	24.25	20.22	0	99
	Ageing population	Percentage of persons aged ≥65 years estimate	19.23	4.80	2.1	57.8
	Lacking fundamental education	Percentage of the persons over 25 years old without a high school diploma estimate	12.40	6.04	1.4	78.7
Psychological factors	Poor mental health	Age-adjusted prevalence of mental health not good for ≥14 days among adults	15.73	2.04	8.3	23.3
Socioeconomic factors	Impoverishment	Percentage of persons below poverty estimate	24.49	8.50	0	71
	Uninsured	Age-adjusted prevalence of current lack of health insurance among adults	16.80	6.61	5.7	53.3
Lifestyle factors	Smoking	Age-adjusted prevalence of current smoking^a^ among adults	20.04	4.10	5.8	41.1
	Binge drinking	Age-adjusted prevalence of binge drinking^b^ among adults	17.84	3.02	8.2	27.6
	Sleep deficiency	Age-adjusted prevalence of sleeping <7 h among adults	34.47	3.64	23.8	48.4
	Sedentary behavior	Age-adjusted prevalence of no leisure-time physical activity among adults	25.71	5.19	10.2	47.2

^a^Current smoking is lifetime smoking of ≥100 cigarettes and currently smoking.

^b^Binge drinking is ≥5 drinks for men, or ≥4 drinks for women at a specific occasion in the last 30 days.

### Network analysis of NCD prevalence

**Fig. 2.**
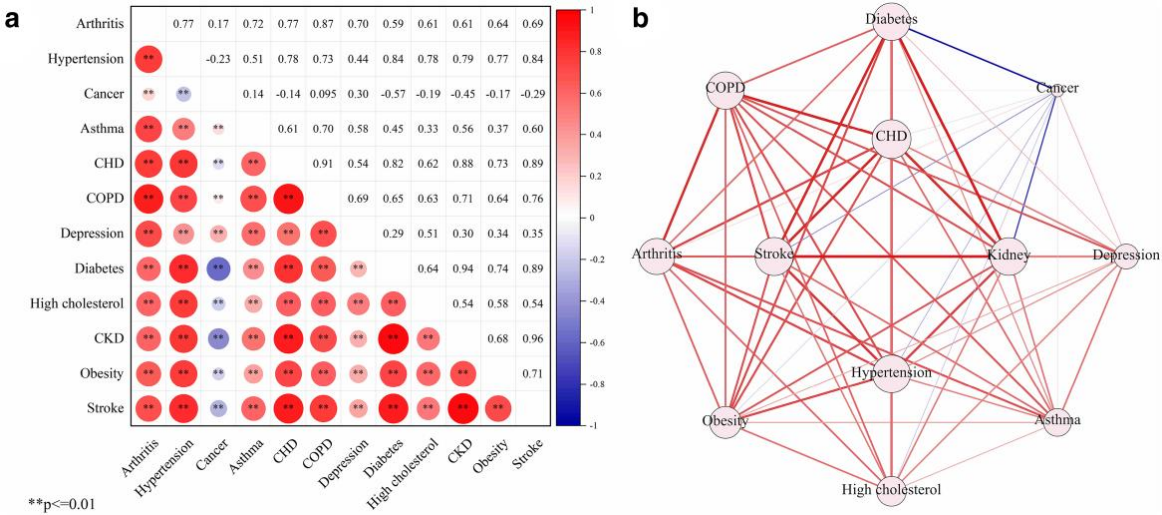
Bivariate interaction analysis of NCD prevalence; a) Pearson correlation heatmap. b) Monomodal network diagram.

Figure 2 depicts the interrelationships among NCDs and identifies MCC pairs. These interconnections reveal relationships among various NCDs. In terms of the strength of disease nodes, CHD exhibits the highest influence, while cancer has the lowest influence (Fig. 2b). Regarding the types of associations between NCDs, cancer shows negative correlations with hypertension, CHD, diabetes, high cholesterol, CKD, obesity, and stroke. Moreover, the study finds the most pronounced negative correlation (−0.57) between cancer and diabetes, whereas the strongest positive correlation (0.96) was found between CKD and stroke. However, there is no significant correlation between cancer and COPD (Fig. 2a).

Table 2 lists the calculation results of disease node importance in the MCC network. The nodes are arranged in descending order of strength: CHD, stroke, hypertension, CKD, diabetes, COPD, arthritis, obesity, high cholesterol, asthma, depression, and cancer. Moreover, the nodes are arranged in descending order of influence: diabetes, CKD, CHD, stroke, hypertension, COPD, arthritis, obesity, high cholesterol, asthma, depression, and cancer. Hypertension, stroke, CHD, CKD, and diabetes exhibit relatively high node strength and disease influence. Conversely, cancer, depression, and asthma show lower levels of both node strength and disease influence.

**Table 2.** Multimorbidity network node calculation results.

NCDs	Node strength	Node influence
Arthritis	7.149	1.1096588
Asthma	5.568	0.8389998
Cancer	2.742	0.404974
CHD	7.696	1.1699424
CKD	7.414	1.1769223
COPD	7.179	1.1250683
Depression	5.043	0.8000157
Diabetes	7.404	1.2024869
High cholesterol	5.958	0.9062387
Hypertension	7.47	1.153484
Obesity	6.357	0.9511319
Stroke	7.571	1.1610772

**Fig. 3.**
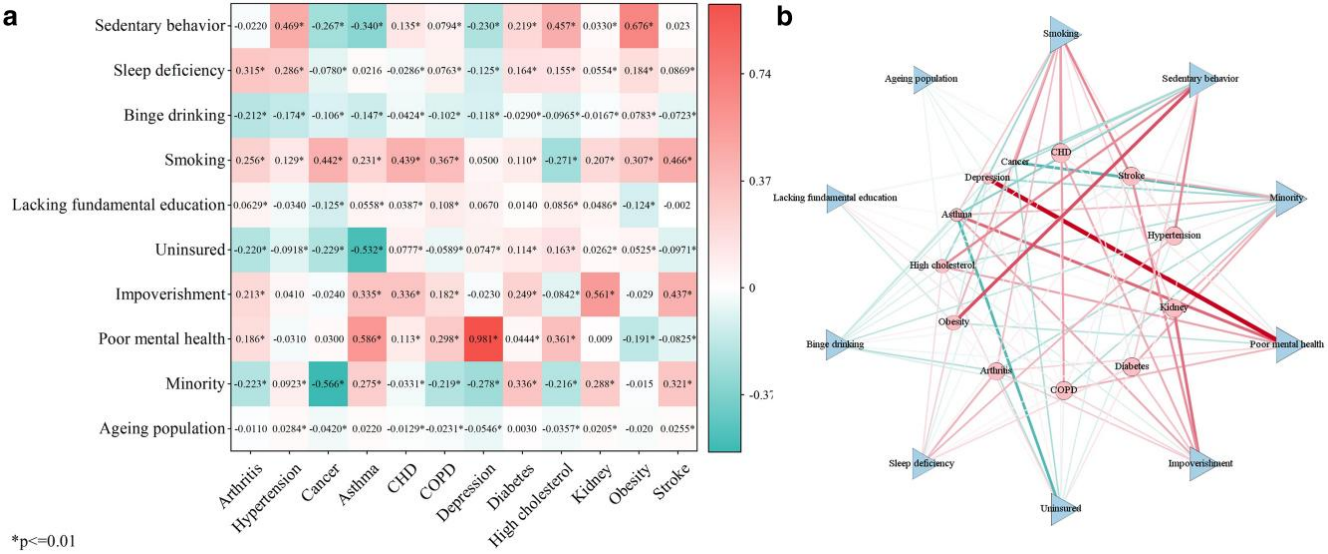
The influence of covariates on the prevalence of NCDs; a) OLS coefficient heatmap. b) Multimodal network diagram.

Figure 3 uses a multimodal network diagram to visually illustrate relationships between NCDs and associated risk factors. The thickness of the lines indicates the magnitude of the regression coefficients in the ordinary least square (OLS) model, emphasizing the substantial impacts of certain factors on the specific disease (Fig. 3a). The significant increase in the prevalence of arthritis, hypertension, CHD, and high cholesterol is significantly associated with “Smoking” and “Poor mental health.” The significant increase in the prevalence of diabetes, stroke, and CKD is connected to factors such as “Minority,” “Sedentary behavior,” and “Smoking.” The prevalence of asthma and COPD shows a significant positive correlation with “Smoking,” “Impoverishment,” “Lacking fundamental education,” and “Poor mental health,” while showing a significant negative correlation with “Uninsured.” The increased prevalence of obesity in the majority of counties is influenced by “Binge drinking,” “Smoking,” and “Sedentary behavior.” The prevalence of depression shows a significant positive correlation with an “Ageing population” and “Poor mental health.” Additionally, risk factors such as “Ageing population” and “Sleep deficiency” have a significant impact on the increasing burden of stroke.

In terms of the impact of risk factors on disease prevalence, the “Smoking” variable is the factor with the largest impact on these NCDs. This implies that if a region has a higher proportion of smokers, the prevalence of these NCDs in the area is likely to be significantly influenced. In contrast, “Ageing population” has a much lower impact on disease prevalence compared with other risk factors, suggesting that although it may be associated with some NCDs, its overall impact at the national level is limited (Fig. 3b).

### MCC pattern mining and analysis

Table 3 summarizes the final community classification results obtained by detecting overlapping community structures in the network. This analysis offers valuable insight into the internal mechanisms and patterns within the MCC network, leading to the identification of four MCC patterns. Specifically, arthritis, asthma, stroke, and hypertension are found to be distributed across multiple communities, signifying their pivotal role in the development and interconnection of MCC.

**Table 3.** Patterns of MCC in NCDs.

Community	NCDs	Risk factors
1	Hypertension, cancer, diabetes, CKD, stroke	Minority, sedentary behavior, smoking
2	Arthritis, asthma, CHD, COPD, high cholesterol, hypertension	Impoverishment, poor mental health, smoking, lacking fundamental education, uninsured
3	Arthritis, asthma, obesity	Smoking, poor mental health, Binge drinking, sedentary behavior
4	Depression, stroke	Poor mental health, ageing population, sleep deficiency

The roles of NCDs in the MCC patterns can be classified by combining the information from Tables 2 and 3. According to the provided definition, we identify CHD, CKD, and diabetes as core diseases due to their high node strength and node influence (Table 2). Arthritis, asthma, stroke, and hypertension are categorized as bridge diseases as they are distributed among multiple MCC patterns. Lastly, cancer, depression, COPD, obesity, and high cholesterol are considered general diseases.

## Discussion

Our research used county-level data from 12 NCDs and up to 10 associated risk factors to explore regional interactions among NCDs and their associations with risk factors. For this purpose, we constructed an integrated MCC network to identify MCC patterns for a scientific classification of NCDs. Consequently, the findings identified four distinct MCC patterns among NCDs.

It should be noted that our network analysis has identified potential comorbidity patterns among NCDs, but these patterns may not directly reflect the actual prevalence of MCC in the general population. The primary objective of this study is to provide a perspective for understanding the comorbidity framework of NCDs and to offer a scientific foundation for public health policy development.

In community 1, we observed a MCC pattern of diabetes-CKD-cancer. Simultaneously, stroke and hypertension showed a bidirectional influence on this pattern. A meta-analytical review indicated a 42% increased risk of kidney cancer associated with diabetes (21), as the kidney is an important organ of glucose homeostasis (22). Previous research has indicated that diabetes places a substantial burden on kidney function. Furthermore, diabetes may potentially increase the risk of developing kidney cancer through its association with hypertension (23, 24). However, our constructed MCC network revealed a surprising negative correlation between cancer and diabetes, as well as hypertension (Fig. 2). A plausible explanation is that the most prevalent cancers in the United States are breast, prostate, lung, and thyroid cancers (25). Patients with these types of cancer exhibit a comparatively lower risk of developing hypertension or diabetes (26–29). This finding underscores the divergent impact of these two NCDs across distinct cancer categories. In addition, results from the MCC network emphasize that the correlation between CKD and stroke is the strongest MCC pattern among all disease combinations. Multiple studies have confirmed that CKD appears to independently increase the risk of stroke by 43%. The association between CKD and stroke may be attributed to the combined influence of conventional and nonconventional cardiovascular mechanisms, with hypertension emerging as the most common complication in CKD patients (30–32). Therefore, the interaction between stroke, hypertension, and CKD has significant implications for the development of this NCDs’ combination. Conversely, we also observed that “Minority,” “Sedentary behavior,” and “Smoking” could be potential risk factors (Fig. 3), introducing fresh directions for exploring the intricate mechanisms of the coexistence among these NCDs. The OLS model illustrates that these factors may promote the occurrence of CKD, stroke, and diabetes. Firstly, race and “Sedentary behavior” have been identified as risk factors for these NCDs (33, 34). Secondly, “Smoking” is considered a shared contributor to both diabetes and kidney cancer (35). It also significantly increases the prevalence of many other NCDs, consistent with previous research results (36). A credible explanation behind this trend is that people who smoke may pay less attention to their personal health. In summary, the impact of CKD and its potential complications should not be underestimated, even though the prevalence of CKD in the United States is quite low (Fig. 1). This may be due to the fact that early CKD is generally asymptomatic, leading to many CKD patients being unaware of their conditions (37). Studies have shown that early detection and improved management of CKD can effectively decelerate its progression (38), playing a critical role in maintaining kidney health and improving cardiovascular health.

Within community 2, we observed two MCC patterns: COPD–asthma and high cholesterol–hypertension–CHD–arthritis. It is noteworthy that there is a significant interaction between these two disease clusters. Research has indicated that over 40% of COPD patients have a history of asthma, with asthma being recognized as a risk factor for the development of COPD (39). The concept of Asthma-COPD overlap syndrome (ACOS) was introduced by the Global Initiative for Asthma in 2014 (40). Furthermore, high cholesterol, hypertension, and arthritis were identified as the most prevalent triad of MCC in the United States (41). Arthritis shows a strong correlation with various chronic conditions. Previous research has shown that arthritis patients often have higher levels of systemic inflammation than nonarthritis patients (42). This inflammation has been identified as a crucial factor in the development of CVD and asthma (43, 44). Besides, hypertension, CHD, and high cholesterol are all encompassed in CVD due to their similar physiological pathogenesis (45). Moreover, COPD and arthritis act as connectors between these two MCC patterns. Patients with COPD tend to be sedentary for long periods of time (46), due to common symptoms such as dyspnea or shortness of breath (47), which can limit physical activities. Consequently, COPD can make arthritis worse if patients lack physical activities (48). On the other hand, we observed that “Uninsured,” “Smoking,” “Lacking fundamental education,” “Impoverishment,” and “Poor mental health” exert varying degrees of influence on the prevalence of these NCDs (Fig. 3). Firstly, compared with patients solely affected by COPD, those with ACOS bear an increased burden of hospitalizations or emergency department visits (49). Therefore, more people choose to purchase insurance to mitigate economic pressures. Secondly, “Smoking” is a recognized risk factor for the development of ACOS (50). Thirdly, previous research has proposed that lower socioeconomic status measured by education and income levels appears to be a risk factor for both asthma and COPD (51). Lastly, smoking and poor mental health are associated with an elevated prevalence of arthritis and CVD (52, 53).

In community 3, we observed a MCC pattern of asthma–obesity, while inflammation stemming from arthritis showed a bidirectional influence on this pattern. Numerous epidemiological studies have elucidated the correlation between obesity and asthma. This correlation is significantly affected by the physiological anomalies commonly shared by both conditions (54). Firstly, obesity leads to an excess accumulation of fat in the chest and abdomen, compressing the lungs and a subsequent reducing in lung capacity (55). This, in turn, triggers or exacerbates the symptoms of asthma (56). Secondly, obesity exacerbates certain inflammatory processes, which affect asthma's severity and management, and cause abnormalities in lung function (57, 58). In conclusion, obesity can directly and indirectly increase the risk of developing arthritis (59). Additionally, the inflammation induced by arthritis is correlated with both asthma and obesity (44, 60). On the other hand, we observed that “Smoking,” “Poor mental health,” “Sedentary behavior,” and “Binge drinking” have an impact on these NCDs (Fig. 3). Firstly, research has suggested that any degree of poor mental health appears to increase the risk of developing asthma (61). Meanwhile, individuals with asthma or asthma–obesity seem to experience poorer social and mental health and higher rates of tobacco use (62). Secondly, “Sedentary behavior” is also associated with the exacerbation of obesity and asthma. A lack of physical activity has been confirmed to increase the risk of obesity (63). Besides, asthma patients engage in significantly less physical exercise (64). Thirdly, “Binge drinking” increases the risk of obesity as drinkers often socialize and dine out more frequently. This behavior may lead to increased consumption of high-salt, high-fat, high-calorie foods (65), which have a negative impact on asthma (54). It is noteworthy that the data for the year 2020 indicate that obesity had the highest prevalence (Fig. 1). This underscores the importance of obesity as a pressing public health issue in the United States and suggests that obesity may potentially become the leading cause of increased prevalence of NCDs such as CVD and diabetes in the future.

In community 4, we observed a MCC pattern of depression-stroke. Current research revealed that post-stroke depression occurs in approximately half of stroke patients (66). Acute stroke is a stressful event that increases the secretion of glucocorticoids, which in turn leads to neurotransmitter abnormalities and increases the risk of depression (67). Additionally, massive meta-analysis results have shown that the risk of first-time stroke in the general population was increased by 40% due to depression (68). This occurs because depression may hinder the pursuit of treatment in patients with MCC, such as promptly seeking medical attention, adhering to medication regimens, and adopting healthy behaviors (69). These negative behaviors will raise the susceptibility to comorbidities in these NCDs. Therefore, the treatment of depression should be considered as a crucial aspect of stroke prevention. On the other hand, we observed that “Poor mental health,” “Ageing population,” and “Sleep deficiency” have different degrees of influence on the prevalence of these NCDs (Fig. 3). Firstly, research demonstrated that younger stroke survivors were more likely to experience depressive symptoms (70). Results from OLS show a particularly strong negative association between the prevalence of depression and the proportion of the population aged ≥65 years, aligning with the findings of several studies (71). However, the clustering risk of stroke with depression shows a tendency to escalate with advancing age (72). This phenomenon may be explained by the fact that younger individuals often experience high levels of employment and parenting demands which in turn may increase their risk of developing depression, this interpretation aligns with the understanding that various stressors, including those related to employment and familial responsibilities, can collectively impact mental health outcomes (73). Moreover, research shows that older individuals may be more accepting of the inevitability of physical illnesses compared with younger individuals, potentially buffering the negative effects of physical illnesses on depression (74). Secondly, numerous pieces of evidence suggested an intricate relationship between stroke and sleep (75, 76). A shorter duration of sleep before a stroke has been associated with an increased likelihood of post-stroke depression (77). Since stroke can disrupt the central nervous system, it often leads to changes in brain activity, brain function, and sleep structure (78).

The findings of this study reveal that NCDs like arthritis, asthma, stroke, and hypertension are prevalent in multiple communities. Through an assessment of node significance, we have identified CHD, CKD, and diabetes as exhibiting both strong node strength and significant impact on disease. Accordingly, we classify CHD, CKD, and diabetes as core diseases. Besides, arthritis, asthma, stroke, and hypertension are categorized as bridge diseases.

Our findings are consistent with existing literature describing MCC at the individual level. Ward and Schiller used the National Health Interview Survey to examine common dyad and triad combinations of ten diseases, highlighting hypertension–arthritis as the most common dyadic combination (3), which is similar to the patterns observed in our identified comorbidity community 2. Additionally, Schiltz provided significant insights into the prevalence and outcomes associated with specific MCC patterns through individual-level survey data. Their research, which represents one of the most comprehensive population-based studies in the United States to date, cataloged 223 distinct chronic disease patterns, including 74 dyadic, 115 triadic, and 34 quadric combinations (79). When we compared their findings to the four comorbidity communities we identified, our results showed a high degree of concordance with the top 50% of specific MCC combinations ranked in Schiltz's paper.

However, it's essential to acknowledge certain limitations inherent in our research. Firstly, all the analyses in this study are limited by the threat of ecological fallacies. Because we use aggregated variables at the county level, without accounting for individual-level factors. To mitigate this limitation, future research should conduct analyses at the individual level to confirm if the results are consistent with those obtained at the county level. Secondly, even though the analysis indicates an association between specific risk factors such as “Smoking” and “Sedentary behavior” with a high prevalence of NCDs, the findings cannot establish causal relationships. NCDs are intricate and interrelated, resulting from a combination of environmental, genetic, and other factors. With the continuous advancement of data mining techniques and the availability of various types of data sources such as remote sensing images, environmental auditing, and sample survey, future research should explore methods to integrate these datasets with the statistical data we used in the study. This integration could facilitate the simulation of residents’ living environments, genetic histories, lifestyles, and other relevant information. Furthermore, the application of data-driven methods could offer a more accurate understanding of the underlying causes of NCDs. Lastly, it's important to note that our analysis did not account for time lag effects. NCDs often result from long-term exposure to risk factors, exhibiting lagged and long-term characteristics. To remedy this limitation, future research should involve longitudinal analysis into the current methodology framework. In the future, we intend to use the Generalized Linear Mixed model to explore the associations between regional human, social, and economic disparities and the spatiotemporal heterogeneity of NCDs. Additionally, considering that county-level data may exhibit significant spatial effects in their distribution, future research could combine spatial analysis with network analysis. Specifically, the distribution patterns of MCC in the population can be explored by calculating the Local Colocation Quotient (80). This multidimensional approach could capture the mechanisms of disease clustering and reveal the co-occurrence relationships of NCDs in different geographic areas.

## Conclusion

In summary, this comprehensive nationwide study of the US adult population has yielded crucial insights into up to 12 NCDs and 10 risk factors related to MCC. It is noteworthy that this subject remains relatively unexplored in existing research. The primary objective of this study is to explore the interrelationships between NCDs and risk factors contributing to the occurrence of MCC in a region. Therefore, two distinct networks were established for this purpose: one dedicated to disease–disease associations, and the other to disease–influencer associations. Subsequently, these networks were integrated into a MCC network, which was then subjected to a thorough network analysis. Ultimately, the NCDs were classified according to their specific roles in the integrated MCC network.

In the present study, we reported that a specific distribution pattern in the correlation of NCDs and various risk factors has different effects on the patterns of MCC. These findings not only hold practical implications for guiding NCDs prevention and treatment but also provide valuable insights for developing improved medical and intervention strategies. Firstly, from the perspective of cost-effectiveness, our results can assist policymakers in optimizing resource allocation. Strategic healthcare workforce planning, precise hospital resource allocation, and targeted research funding are imperative for populations at the highest risk of MCC, thereby alleviating the economic burden. Secondly, improving patient care and health outcomes is imperative. Recognizing the interrelationships between diseases can significantly enhance disease management and empower healthcare professionals to craft holistic treatment plans, yielding better patient outcomes. Thirdly, governments should pay more attention to preventive measures and early intervention. Identifying shared risk factors and root causes plays a pivotal role in preventing and addressing chronic diseases proactively. Fourthly, governments should elevate public awareness of MCC by educating patients on the intricate connections between NCDs and encouraging them to play a more active role in their healthcare management. Lastly, our results can inform governments in refining healthcare policies and promoting health equity. Policymakers can leverage data on MCC patterns and risk factors to shape evidence-based healthcare policies.

This research significantly advances our understanding of MCC and represents a substantial breakthrough in quantifying the burden of multiple NCDs. Moreover, it offers a foundation for developing a more harmonized and standardized approach to addressing the various types of MCC. Our objective is to apply comparable methods to address other public health challenges in different countries.

## Materials and methods

### Data

We performed a secondary data analysis of the nationwide prevalence of NCDs using county-level population health outcome data from the 2020 PLACES project in the United States. This dataset estimates age-adjusted NCD prevalence for 3,143 counties through a small area estimation approach, derived from state-level individual data provided by the Behavioral Risk Factor Surveillance System (BRFSS). The BRFSS is a large monitoring dataset on behavioral risk factors collected by the Centers for Disease Control and Prevention (CDC). The CDC utilized a complex sampling design that included both landline and cell phone respondents to ensure a representative sample of the US population. These data are based on self-reports from respondents and include answers to questions about smoking, drinking, physical activity, and health-related lifestyles. Compared with nationwide survey results, these data offer higher reliability and accuracy (81). The PLACES project data we analyzed expands the geographic coverage of the BRFSS from the state level to the county level, encompassing respondents aged 18 and older from all 50 states and 3,143 counties in the United States. This study focused on 11 chronic conditions (including arthritis, hypertension, cancer, asthma, CHD, COPD, depression, diabetes, high cholesterol, CKD, and stroke) from a list of 20 identified by the US Department of Health and Human Services (HHS). This selection ensures a consistent and standardized approach to measuring the occurrence of chronic conditions (82). Additionally, we also included obesity in our analysis of MCC due to its widespread prevalence nationwide and the accessibility of its data. Besides, obesity aligns with the HHS definition of a NCD as “conditions that last a year or more and require ongoing medical attention and/or limit activities of daily living (82).” Figure 1 depicts the distribution of prevalence data (age-adjusted) for these NCDs, the vertical axis represents prevalence rates, and the plot's area size indicates the number of US counties falling within specific prevalence rate ranges.

Previous studies have reported that NCDs are affected by a variety of biological, psychological, and social factors (4). As a result, we selected a total of 10 explanatory variables related to the prevalence of NCDs (10, 17, 83–86). These variables cover biological, psychological, socioeconomic, and behavioral aspects, representing potential risk factors for NCDs. These covariates are sourced from various data repositories, including the Social Vulnerability Index (SVI) database and the PLACES database. More precisely, the SVI database provides data on three biological factor variables and economic factors within socio-environmental parameters. These data are summarized based on the American Community Survey 2017–2021 or 2016–2020 estimates. Furthermore, the PLACES database provides information on lifestyle behavioral factors, health status data, and socio-environmental factors measures, using small-area estimation methods.

Considering the outbreak of the COVID-19 pandemic in March 2020, the dataset used in this study includes data from both before and after the pandemic outbreak, as it does not break down to the specific month of collection. This cross-sectional analysis is limited to the 2020 dataset. To evaluate the pandemic's potential influence, we additionally collected data for 2019 and 2021. By comparing the prevalence of NCDs across three years, we found no significant differences in the 2020 data. This suggests that the impact of the pandemic on the distribution of the study data was minimal.

The data are collected at the county level from the CDC in the United States. This dataset not only offers a more precise depiction of the distribution trends of various risk factors but also provides essential data support for constructing the network model in subsequent analyses. Table 1 presents the classification and statistical characteristics of the risk factors dataset, where the numbers represent the percentage of individuals relative to the total population.

### MCC network construction

To comprehensively reveal underlying mechanisms of MCC in the United States, we constructed two distinct networks using Pearson correlation analysis and OLS. Subsequently, we amalgamated these networks to form an integrated MCC network model. This model enables the investigation of interactions between NCDs and their associations with risk factors. Through this approach, we explore MCC patterns which offer theoretical and data support for the development of management and prevention strategies for multiple NCDs clusters.

### Monomodal network of disease–disease associations

We utilize Pearson correlation analysis to identify relevant pairs of NCDs. Subsequently, we construct a monomodal network of disease–disease associations based on these results. The network is denoted as *G*  *=* (*D, E_DD_*), where *D* represents the set of NCDs, and *E_DD_* represents the set of edges that signify the degrees of correlations between NCDs. These edges are defined by the adjacency matrix *A = {a_ij_}*, as described in formula (1). The interconnections between NCDs are visually presented by complex network visualization technique to lay the groundwork for identifying MCC patterns.


(1) $$\begin{array}{r} a_{ij}=\{\begin{array}{cc} w_{ij}; & \begin{array}{c} \mathrm{The}\;\mathrm{value}\;\mathrm{of}\;\mathrm{the}\;\mathrm{correlation}\;\mathrm{coefficient}\;\mathrm{between}\;\\ \mathrm{disease}\;i\;\mathrm{and}\;\mathrm{disease}\;j. \end{array}\\ 0; & \mathrm{Disease}\;i\mathrm{and}\;\mathrm{disease}\;j\mathrm{are}\;\mathrm{unrelated}. \end{array} \end{array}\;(1)$$

### Multimodal network of disease–influencer associations

In order to explore the correlation between different NCDs and risk factors across the United States, we employed the OLS model within a multimodal network. In the OLS model, we designated the prevalence of NCDs as the response variable and the risk factors as the explanatory variables. This network encompasses two distinct types of nodes and edges, denoted as *F*  *=* (*D, U, E_DU_*), where *D*, *U*, and *E_DU_* respectively represent the set of NCDs, risk factors, and the value of the regression coefficient obtained from the OLS model.

**Fig. 4.**
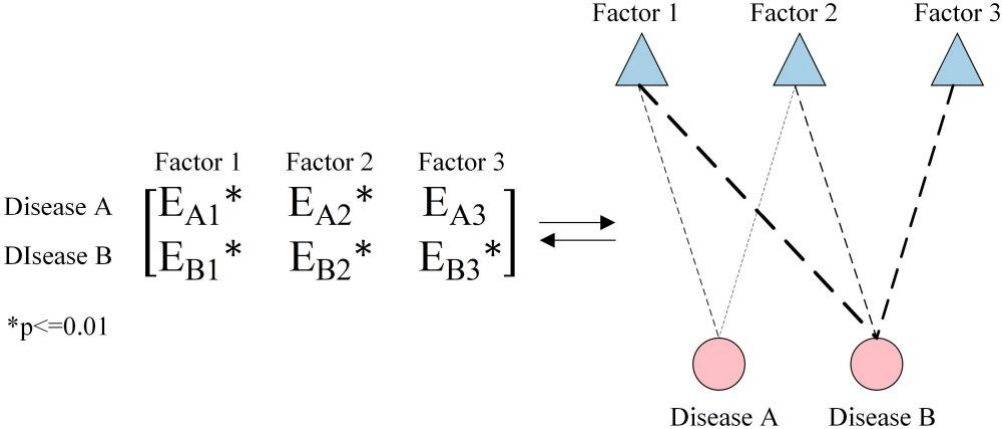
Schematic representation of multimodal network construction.

The multimodal network diagram is illustrated in Fig. 4. It is worth noting that disease nodes are only connected to risk factor nodes, and no connections are established between disease nodes, similarly for influence nodes.

### Integration of MCC network

In order to incorporate the risk factors of NCDs into the contributors of MCC, we merged the two networks that were previously built to construct the integrated MCC network. This network includes two dimensions of relationships: one is the connections between NCDs, and the other is the associations between different NCDs and risk factors. Furthermore, we apply node identification algorithms in the MCC network that make use of recognizing overlapping nodes to reveal community structures characterized by close interconnections and shared attributes. This approach allows for the elucidation of the structural attributes of each community, defining distinct MCC patterns more effectively across distinct population groups.

**Fig. 5.**
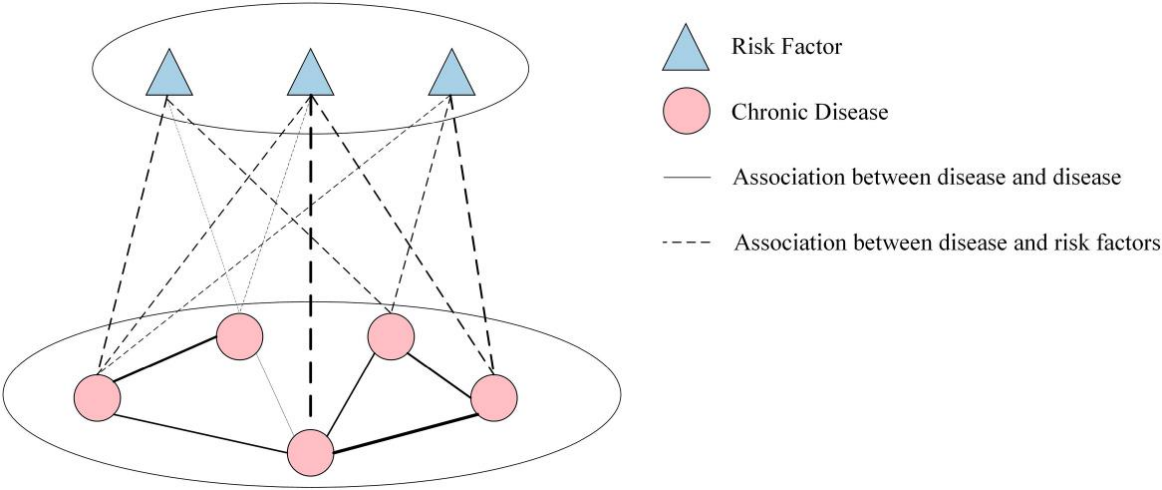
Schematic representation of MCC network construction.

Based on the associations of disease–disease and disease–influencer, we established edges both among NCDs and between NCDs and associated risk factors. As a result, we created an integrated MCC network consisting of sets of disease nodes and risk factor nodes. The MCC network diagram is illustrated in Fig. 5.

### Network analysis of MCC network

#### Overlapping node recognition algorithm

The Local Fitness Maximization (LFM) algorithm can simultaneously discover overlapping communities and hierarchical structures (87). This algorithm assesses the suitability of adding nodes to a local community through a fitness function that measures the rationality of the community partition. We used the LFM algorithm for identifying community structures without sacrificing any data within the integrated MCC network. The implementation process of the LFM is as follows:

Begin by randomly selecting a node that has not been assigned to any community. This selected node is then added as a seed node to form a new community.

Next, we extend the community by iterating through each node *i* in the neighboring node set of the community. For each node, we calculate the difference between the fitness functions of two communities. One community included node *i* (denoted as {*G*  *+*  *i*}), and the other excluded node *i* (denoted as {*G − i*}). We indicate this distinction as *f′ G* = *f_G+i_ − f_G−i_*. Once we calculate this value for all nodes in the set, we add the node with the highest *f′ G* value to the community *G*. Then, we used this newly added node as a seed node. We repeated the process until the fitness function stopped increasing, representing the end of the community *G* expansion. The calculation of the fitness function for community *G* is as shown in formula (2).


(2) $$f_{G}=\frac{k_{\mathrm{in}\;}^{G}}{{\left(k_{\mathrm{in}\;}^{G}+k_{\mathrm{out}\;}^{G}\right)}^{\alpha }}.$$

The variables $k_{\mathrm{in}\;}^{G}$ and $k_{\mathrm{out}\;}^{G}$ respectively represent the internal and external degrees of the community. The internal degree of a community is equal to twice the number of internal edges within the community. While, the external degree is equal to the number of edges connecting nodes in the community to nodes located outside the community. The real number parameter *α* is used to control the size of the communities. Decreasing the value of *α* results in the partitioning of larger-sized communities.

Finally, to enhance the quality of results, we merge similar communities. During the process of community expansion, multiple communities may share many common nodes. In order to optimize the result of overlapping communities, a method is introduced for calculating the similarity *S* between the nodes of the communities (88). Formula (3) defines *S* by considering the proportion of overlapping nodes in both communities and the size of the smaller communities. If the calculated value of *S* is greater than the preset threshold *β*, it would be recommended to merge those communities. This leads to the final result of overlapping community partitioning.


(3) $$S(G_{1},G_{2})=\frac{\left|G_{1}\cap G_{2}\right|}{\min(\left|G_{1}|,|G_{2}\right|)}.$$

In formula (3), the variables *G*_1_ and *G*_2_ represent different communities. The variable |*G*_1_  *∩*  *G*_2_| indicates the number of overlapping nodes that are shared between *G*_1_ and *G*_2_. |*G*_1_| and |*G*_2_| respectively represent the total number of nodes in *G*_1_ and *G*_2_. Additionally, min(|*G*_1_  *∩*  *G*_2_|) stands for the minimum number of nodes between *G*_1_ and *G*_2_.

#### Nodal importance analysis

In complex networks, a node's influence is not only determined by its own weight but also by the influence of its neighboring nodes. We introduce two metrics to analyze the importance of network nodes.

The first metric is node strength. It is defined as the sum of the weights of all edges connected to the node. The calculation method is shown in formula (4).


(4) $$D_{i}=\sum_{\;j\in \Gamma (i)}A_{ij}.$$

In formula (4), *D_i_* represents the degree of node *i*. *A_ij_* represents the weight of the edge connecting nodes *i* and *j*. *Γ*(*i*) represents the set of neighboring nodes of node *i*.

The second metric is node influence. It is defined as the influence of a node on the surrounding network. The calculation method is shown in formula (5).


(5) $$NI_{i}=\sum_{\;j\in \Gamma (i)}\frac{A_{ij}}{D_{j}}.$$

**Fig. 6.**
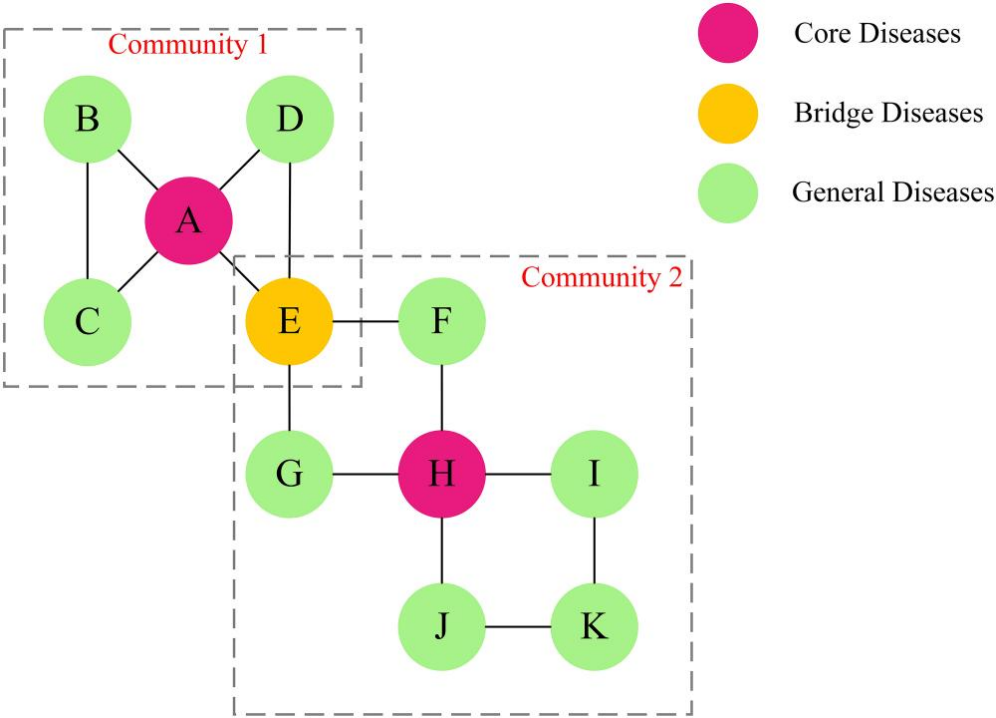
Conceptual mapping of disease roles in MCC networks.

We intend to define and categorize disease roles by evaluating the significance of disease nodes within the MCC network (Fig. 6). This classification enhances our understanding of the relationships between NCDs and provides valuable insights into the evolution and prevention of these diseases. Specifically, NCDs are classified into three distinct groups based on the evaluation of node strength, node influence, and community partitioning results. *Core diseases* are characterized by their large connection sizes and high influence within the MCC network. They typically involve a substantial portion of the disease nodes in their respective communities. As crucial components of the overall disease network, core diseases play a critical role in its functioning. *Bridge diseases* belong to multiple communities in the MCC network. Although their connection sizes are smaller than those of the core diseases, they are instrumental in facilitating the development of MCC across communities by serving as connectors between different communities. *General diseases* are characterized by relatively small connection sizes and influence within the MCC network. They generally do not belong to multiple communities and only connect to a limited fraction of nodes or to nodes with lower node strengths in the network.

## Data Availability

All data and materials have been uploaded and are available via Zenodo: https://doi.org/10.5281/zenodo.14059660.

## References

[1] Beaglehole R, Yach D. 2003. Globalisation and the prevention and control of non-communicable disease: the neglected chronic diseases of adults. Lancet. 362:903–908. 13678979 10.1016/S0140-6736(03)14335-8

[2] Koroukian SM, Warner DF, Owusu C, Given CW. 2015. Multimorbidity redefined: prospective health outcomes and the cumulative effect of co-occurring conditions. Prev Chronic Dis. 12:140478. 10.5888/pcd12.140478 PMC4415428 25906436

[3] Boersma P, Black LI, Ward BW. 2020. Prevalence of multiple chronic conditions among US adults, 2018. Prev Chronic Dis. 17:E106. 32945769 10.5888/pcd17.200130 PMC7553211

[4] Singer M, Bulled N, Ostrach B, Mendenhall E. 2017. Syndemics and the biosocial conception of health. Lancet. 389:941–950. 28271845 10.1016/S0140-6736(17)30003-X

[5] Hajat C, Stein E. 2018. The global burden of multiple chronic conditions: a narrative review. Prev Med Rep. 12:284–293. 30406006 10.1016/j.pmedr.2018.10.008 PMC6214883

[6] Satariano WA, Boyd CM. 2013. Improving the evidence base on multimorbidities through better research: a commentary on the U.S. HHS initiative, multiple chronic conditions: a strategic framework. J. Comorbidity. 3:18–21. PMC5636030 29090142

[7] Shin EK, Kwon Y, Shaban-Nejad A. 2019. Geo-clustered chronic affinity: pathways from socio-economic disadvantages to health disparities. JAMIA Open. 2:317–322. 31984364 10.1093/jamiaopen/ooz029 PMC6951975

[8] American Geriatrics Society Expert Panel on the Care of Older Adults with Multimorbidity . 2012. Guiding principles for the care of older adults with multimorbidity: an approach for clinicians. J Am Geriatr Soc. 60:E1–E25. 22994865 10.1111/j.1532-5415.2012.04188.x PMC4450364

[9] McPhail SM . 2016. Multimorbidity in chronic disease: impact on health care resources and costs. Risk Manag Healthc Policy. 9:143–156. 27462182 10.2147/RMHP.S97248 PMC4939994

[10] Singh K, et al 2019. Multimorbidity in South Asian adults: prevalence, risk factors and mortality. J Public Health. 41:80–89. 10.1093/pubmed/fdy017 PMC7304513 29425313

[11] The Emerging Risk Factors Collaboration . 2010. Diabetes mellitus, fasting blood glucose concentration, and risk of vascular disease: a collaborative meta-analysis of 102 prospective studies. Lancet. 375:2215–2222. 20609967 10.1016/S0140-6736(10)60484-9 PMC2904878

[12] Tinetti ME, et al 2011. Contribution of multiple chronic conditions to universal health outcomes. J Am Geriatr Soc. 59:1686–1691. 21883118 10.1111/j.1532-5415.2011.03573.x PMC3622699

[13] Zemedikun DT, Gray LJ, Khunti K, Davies MJ, Dhalwani NN. 2018. Patterns of multimorbidity in middle-aged and older adults: an analysis of the UK biobank data. Mayo Clin Proc. 93:857–866. 29801777 10.1016/j.mayocp.2018.02.012

[14] Suls J, et al 2022. Emerging approaches to multiple chronic condition assessment. J Am Geriatr Soc. 70:2498–2507. 35699153 10.1111/jgs.17914 PMC9489607

[15] Chen Y, Li L, Xu R. 2015. Disease comorbidity network guides the detection of molecular evidence for the link between colorectal cancer and obesity. AMIA Jt Summits Transl Sci Proc. 2015:201–206. 26306270 PMC4525229

[16] Birk JL, et al 2019. Depression and multimorbidity: considering temporal characteristics of the associations between depression and multiple chronic diseases. Health Psychol. 38:802–811. 31008648 10.1037/hea0000737 PMC6706317

[17] Liu X, Song F, Liu F, Mao Z, Qu S. 2022. Multiple chronic conditions among older adults in China: differences in socio-demographic characteristics. Heliyon. 8:e11129. 36281412 10.1016/j.heliyon.2022.e11129 PMC9586908

[18] Vo A, Tao Y, Li Y, Albarrak A. 2023. The association between social determinants of health and population health outcomes: ecological analysis. JMIR Public Health Surveill. 9:e44070. 36989028 10.2196/44070 PMC10131773

[19] Chukwusa E, Font-Gilabert P, Manthorpe J, Healey A. 2023. The association between social care expenditure and multiple-long term conditions: a population-based area-level analysis. J. Multimorb. Comorbidity. 13:26335565231208994. 10.1177/26335565231208994 PMC10612455 37900010

[20] Hong Y-R, Mainous AG 3rd. 2020. Development and validation of a county-level social determinants of health risk assessment tool for cardiovascular disease. Ann Fam Med. 18:318–325. 32661032 10.1370/afm.2534 PMC7358032

[21] Bao C, et al 2013. Diabetes mellitus and incidence and mortality of kidney cancer: a meta-analysis. J Diabetes Complications. 27:357–364. 23433629 10.1016/j.jdiacomp.2013.01.004

[22] Ruan X, Guan Y. 2009. Metabolic syndrome and chronic kidney disease. J Diabetes. 1:236–245. 20923524 10.1111/j.1753-0407.2009.00042.x

[23] Chow W-H, Gridley G, Fraumeni JF Jr, Jarvholm B. 2000. Obesity, hypertension, and the risk of kidney cancer in men. N Engl J Med. 343:1305–1311. 11058675 10.1056/NEJM200011023431804

[24] Epstein M, Sowers JR. 1992. Diabetes-mellitus and hypertension. Hypertension. 19:403–418. 1568757 10.1161/01.hyp.19.5.403

[25] Rahib L, et al 2014. Projecting cancer incidence and deaths to 2030: the unexpected burden of thyroid, liver, and pancreas cancers in the United States. Cancer Res. 74:2913–2921. 24840647 10.1158/0008-5472.CAN-14-0155

[26] Schoormans D, et al 2018. Incidence of cardiovascular disease up to 13 year after cancer diagnosis: a matched cohort study among 32 757 cancer survivors. Cancer Med. 7:4952–4963. 30220107 10.1002/cam4.1754 PMC6198235

[27] Strongman H, et al 2019. Medium and long-term risks of specific cardiovascular diseases in survivors of 20 adult cancers: a population-based cohort study using multiple linked UK electronic health records databases. Lancet. 394:1041–1054. 31443926 10.1016/S0140-6736(19)31674-5 PMC6857444

[28] Pierce BL . 2012. Why are diabetics at reduced risk for prostate cancer? A review of the epidemiologic evidence. Urol Oncol. 30:735–743. 23021557 10.1016/j.urolonc.2012.07.008

[29] Sona MF, Myung S-K, Park K, Jargalsaikhan G. 2018. Type 1 diabetes mellitus and risk of cancer: a meta-analysis of observational studies. Jpn J Clin Oncol. 48:426–433. 29635473 10.1093/jjco/hyy047

[30] Saland JM, et al 2019. Change in dyslipidemia with declining glomerular filtration rate and increasing proteinuria in children with CKD. Clin J Am Soc Nephrol. 14:1711–1718. 31712386 10.2215/CJN.03110319 PMC6895497

[31] Toyoda K, Ninomiya T. 2014. Stroke and cerebrovascular diseases in patients with chronic kidney disease. Lancet Neurol. 13:823–833. 25030514 10.1016/S1474-4422(14)70026-2

[32] Muntner P, et al 2010. Hypertension awareness, treatment, and control in adults with CKD: results from the Chronic Renal Insufficiency Cohort (CRIC) study. Am J Kidney Dis. 55:441–451. 19962808 10.1053/j.ajkd.2009.09.014 PMC2866514

[33] Dreyer G, Hull S, Mathur R, Chesser A, Yaqoob MM. 2013. Progression of chronic kidney disease in a multi-ethnic community cohort of patients with diabetes mellitus. Diabet Med. 30:956–963. 23600455 10.1111/dme.12197

[34] Cao Z, Xu C, Zhang P, Wang Y. 2022. Associations of sedentary time and physical activity with adverse health conditions: outcome-wide analyses using isotemporal substitution model. eClinicalMedicine. 48:101424. 35516443 10.1016/j.eclinm.2022.101424 PMC9065298

[35] Willi C, Bodenmann P, Ghali WA, Faris PD, Cornuz J. 2007. Active smoking and the risk of type 2 diabetes—a systematic review and meta-analysis. JAMA. 298:2654–2664. 18073361 10.1001/jama.298.22.2654

[36] Ambrose JA, Barua RS. 2004. The pathophysiology of cigarette C-V smoking and cardiovascular disease—an update. J Am Coll Cardiol. 43:1731–1737. 15145091 10.1016/j.jacc.2003.12.047

[37] Brown WW, et al 2003. Early detection of kidney disease in community settings: the Kidney Early Evaluation Program (KEEP). Am J Kidney Dis. 42:22–35. 12830453 10.1016/s0272-6386(03)00405-0

[38] Fragala MS, Shiffman D, Birse CE. 2019. Population health screenings for the prevention of chronic disease progression. Am J Manag Care. 25:548–553. 31747233

[39] Hersh CP, Jacobson FL, Gill R, Silverman EK. 2007. Computed tomography phenotypes in severe, early-onset chronic obstructive pulmonary disease. COPD. 4:331–337. 18027160 10.1080/15412550701601274

[40] Postma DS, Rabe KF. 2015. The asthma-COPD overlap syndrome. N Engl J Med. 373:1241–1249. 26398072 10.1056/NEJMra1411863

[41] Newman D, Tong M, Levine E, Kishore S. 2020. Prevalence of multiple chronic conditions by U.S. state and territory, 2017. PLoS One. 15:e0232346. 32369509 10.1371/journal.pone.0232346 PMC7199953

[42] Sattar N, McInnes IB. 2005. Vascular comorbidity in rheumatoid arthritis: potential mechanisms and solutions. Curr Opin Rheumatol. 17:286–292. 15838238 10.1097/01.bor.0000158150.57154.f9

[43] Ferrucci L, Fabbri E. 2018. Inflammageing: chronic inflammation in ageing, cardiovascular disease, and frailty. Nat Rev Cardiol. 15:505–522. 30065258 10.1038/s41569-018-0064-2 PMC6146930

[44] Ishmael FT . 2011. The inflammatory response in the pathogenesis of asthma. J Am Osteopath Assoc. 111:S11–S17. 22162373

[45] Mendis S, Puska P, Norrving BE, World Health Organization. 2011. Global atlas on cardiovascular disease prevention and control. World Health Organization.

[46] McKeough ZJ, Large SL, Spencer LM, Cheng SWM, McNamara RJ. 2020. An observational study of self-reported sedentary behaviour in people with chronic obstructive pulmonary disease and bronchiectasis. Braz J Phys Ther. 24:399–406. 31182285 10.1016/j.bjpt.2019.05.005 PMC7564013

[47] Spruit MA, et al 2013. An official American Thoracic Society/European Respiratory Society statement: key concepts and advances in pulmonary rehabilitation. Am J Respir Crit Care Med. 188:E13–E64. 24127811 10.1164/rccm.201309-1634ST

[48] Booth FW, Roberts CK, Laye MJ. 2012. Lack of exercise is a major cause of chronic diseases. Compr Physiol. 2:1143–1211. 23798298 10.1002/cphy.c110025 PMC4241367

[49] Kumbhare S, Pleasants R, Ohar JA, Strange C. 2016. Characteristics and prevalence of asthma/chronic obstructive pulmonary disease overlap in the United States. Ann Am Thorac Soc. 13:803–810. 26974689 10.1513/AnnalsATS.201508-554OC

[50] Chiba S, et al 2017. Interstitial changes in asthma-COPD overlap syndrome. Clin Respir J. 11:1024–1031. 26833590 10.1111/crj.12461

[51] Kanervisto M, et al 2011. Low socioeconomic status is associated with chronic obstructive airway diseases. Respir Med. 105:1140–1146. 21459567 10.1016/j.rmed.2011.03.008

[52] Rao Y, et al 2018. Health-related quality of life in patients with arthritis: a cross-sectional survey among middle-aged adults in Chongqing, China. Int J Environ Res Public Health. 15:768. 29659544 10.3390/ijerph15040768 PMC5923810

[53] Bays HE, et al 2021. Ten things to know about ten cardiovascular disease risk factors. Am J Prev Cardiol. 5:100149. 34327491 10.1016/j.ajpc.2021.100149 PMC8315386

[54] Peters U, Dixon A, Forno E. 2018. Obesity and asthma. J Allergy Clin Immunol. 141:1169–1179. 29627041 10.1016/j.jaci.2018.02.004 PMC5973542

[55] Watson R, et al 2010. Reduction of total lung capacity in obese men: comparison of total intrathoracic and gas volumes. J Appl Physiol (1985). 108:1605–1612. 20299612 10.1152/japplphysiol.01267.2009 PMC2886677

[56] Kuder MM, Nyenhuis SM. 2020. Optimizing lifestyle interventions in adult patients with comorbid asthma and obesity. Ther Adv Respir Dis. 14:1753466620906323. 32103702 10.1177/1753466620906323 PMC7047422

[57] Rastogi D, et al 2015. Inflammation, metabolic dysregulation, and pulmonary function among obese urban adolescents with asthma. Am J Respir Crit Care Med. 191:149–160. 25457349 10.1164/rccm.201409-1587OC PMC4347436

[58] Desai D, et al 2013. Elevated sputum interleukin-5 and submucosal eosinophilia in obese individuals with severe asthma. Am J Respir Crit Care Med. 188:657–663. 23590263 10.1164/rccm.201208-1470OC PMC3826183

[59] Blueher M . 2019. Obesity: global epidemiology and pathogenesis. Nat Rev Endocrinol. 15:288–298. 30814686 10.1038/s41574-019-0176-8

[60] Ebron K, et al 2015. A larger body mass index is associated with increased atherogenic dyslipidemia, insulin resistance, and low-grade inflammation in individuals with metabolic syndrome. Metab Syndr Relat Disord. 13:458–464. 26431271 10.1089/met.2015.0053

[61] Chun TH, Weitzen SH, Fritz GK. 2008. The asthma/mental health nexus in a population-based sample of the United States. Chest. 134:1176–1182. 18719055 10.1378/chest.08-1528

[62] Hawkins MAW, et al 2020. Psychological distress and substance use among young adults with comorbid asthma and obesity. J Am Coll Health. 68:914–921. 31373892 10.1080/07448481.2019.1643353

[63] Esparza J, et al 2000. Daily energy expenditure in Mexican and USA Pima Indians: low physical activity as a possible cause of obesity. Int J Obes Relat Metab Disord. 24:55–59. 10702751 10.1038/sj.ijo.0801085

[64] Teramoto M, Moonie S. 2011. Physical activity participation among adult Nevadans with self-reported asthma. J Asthma. 48:517–522. 21486198 10.3109/02770903.2011.567426

[65] Ni W, et al 2019. Clustering of cardiovascular disease biological risk factors among older adults in Shenzhen City, China: a cross-sectional study. BMJ Open. 9:e024336. 10.1136/bmjopen-2018-024336 PMC6429887 30850407

[66] Ayerbe L, Ayis S, Wolfe CDA, Rudd AG. 2013. Natural history, predictors and outcomes of depression after stroke: systematic review and meta-analysis. Br J Psychiatry. 202:14–21. 23284148 10.1192/bjp.bp.111.107664

[67] Van den Berghe G, Schoonheydt K, Becx P, Bruyninckx F, Wouters PJ. 2005. Insulin therapy protects the central and peripheral nervous system of intensive care patients. Neurology. 64:1348–1353. 15851721 10.1212/01.WNL.0000158442.08857.FC

[68] Barlinn K, et al 2014. Exploring the risk-factor association between depression and incident stroke: a systematic review and meta-analysis. Neuropsychiatr Dis Treat. 11:1–14. 25565846 10.2147/NDT.S63904 PMC4274141

[69] Penninx BW, et al 1998. Depressive symptoms and physical decline in community-dwelling older persons. JAMA. 279:1720–1726. 9624025 10.1001/jama.279.21.1720

[70] McCarthy MJ, et al 2016. Age, subjective stress, and depression after ischemic stroke. J Behav Med. 39:55–64. 26245159 10.1007/s10865-015-9663-0 PMC4724284

[71] Jungehülsing GJ, et al 2008. Prevalence of stroke and stroke symptoms: a population-based survey of 28,090 participants. Neuroepidemiology. 30:51–57. 18259083 10.1159/000115750

[72] Weinberger AH, et al 2018. Trends in depression prevalence in the USA from 2005 to 2015: widening disparities in vulnerable groups. Psychol Med. 48:1308–1315. 29021005 10.1017/S0033291717002781

[73] Moen P, et al 2015. Is work-family conflict a multilevel stressor linking job conditions to mental health? Evidence from the work, family and health network. Res Sociol Work. 26:177–217. 25866431 10.1108/S0277-283320150000026014 PMC4389766

[74] Ernst C, Angst J. 1995. Depression in old age. Is there a real decrease in prevalence? A review. Eur Arch Psychiatry Clin Neurosci. 245:272–287. 8527464 10.1007/BF02191869

[75] Fang J, Wheaton AG, Ayala C. 2014. Sleep duration and history of stroke among adults from the USA. J Sleep Res. 23:531–537. 24815229 10.1111/jsr.12160 PMC4365417

[76] Ruiter Petrov ME, Letter AJ, Howard VJ, Kleindorfer D. 2014. Self-reported sleep duration in relation to incident stroke symptoms: nuances by body mass and race from the REGARDS study. J Stroke Cerebrovasc Dis. 23:e123–e132. 24119626 10.1016/j.jstrokecerebrovasdis.2013.09.009 PMC3946730

[77] Dong L, et al 2021. Pre-stroke sleep duration and post-stroke depression. Sleep Med. 77:325–329. 32828696 10.1016/j.sleep.2020.04.025 PMC7667889

[78] Pasic Z, Smajlovic D, Dostovic Z, Kojic B, Selmanovic S. 2011. Incidence and types of sleep disorders in patients with stroke. Med Arh. 65:225–227. 21950229 10.5455/medarh.2011.65.225-227

[79] Schiltz NK . 2022. Prevalence of multimorbidity combinations and their association with medical costs and poor health: a population-based study of U. S. adults. Front Public Health. 10:953886. 36466476 10.3389/fpubh.2022.953886 PMC9717681

[80] Cromley EK, Wilson-Genderson M, Heid AR, Pruchno RA. 2018. Spatial associations of multiple chronic conditions among older adults. J Appl Gerontol. 37:1411–1435. 27697796 10.1177/0733464816672044

[81] Pierannunzi C, Hu SS, Balluz L. 2013. A systematic review of publications assessing reliability and validity of the Behavioral Risk Factor Surveillance System (BRFSS), 2004–2011. BMC Med Res Methodol. 13:49. 23522349 10.1186/1471-2288-13-49 PMC3622569

[82] Goodman RA, Posner SF, Huang ES, Parekh AK, Koh HK. 2013. Defining and measuring chronic conditions: imperatives for research, policy, program, and practice. Prev Chronic Dis. 10:E66. 23618546 10.5888/pcd10.120239 PMC3652713

[83] Adams ML, Grandpre J, Katz DL, Shenson D. 2017. Linear association between number of modifiable risk factors and multiple chronic conditions: results from the Behavioral Risk Factor Surveillance System. Prev Med. 105:169–175. 28917949 10.1016/j.ypmed.2017.09.013

[84] Assari S, Lankarani MM. 2014. Race and ethnic differences in the associations between cardiovascular diseases, anxiety, and depression in the United States. Int J Travel Med Glob Health. 2:107–113. 31396543 PMC6687331

[85] Prince MJ, et al 2015. The burden of disease in older people and implications for health policy and practice. Lancet. 385:549–562. 25468153 10.1016/S0140-6736(14)61347-7

[86] Jerant AF, von Friederichs-Fitzwater MM, Moore M. 2005. Patients’ perceived barriers to active self-management of chronic conditions. Patient Educ Couns. 57:300–307. 15893212 10.1016/j.pec.2004.08.004

[87] Lancichinetti A, Fortunato S, Kertesz J. 2009. Detecting the overlapping and hierarchical community structure in complex networks. New J Phys. 11:033015.

[88] Yu Z-Y, Chen J-J, Guo K, Chen Y-Z, Xu Q. 2019. Overlapping community detection based on influence and seeds extension. Acta Electron Sin. 47:153–160.

